# Implementation of a Statewide Surveillance System for Neonatal Abstinence Syndrome — Tennessee, 2013

**Published:** 2015-02-13

**Authors:** Michael D. Warren, Angela M. Miller, Julie Traylor, Audrey Bauer, Stephen W. Patrick

**Affiliations:** 1 Tennessee Department of Health; 2 Department of Pediatrics and Health Policy, Division of Neonatology, Vanderbilt University School of Medicine

Over the last decade, rates of opioid pain reliever prescribing grew substantially in the United States, affecting many segments of the population, including pregnant women (1). Nationally, Tennessee ranks second in the rate of prescriptions written for opioid pain relievers, with 1.4 per person in 2012 (2). The rising prevalence of opioid pain reliever use and misuse in Tennessee led to an increase in adverse outcomes in the state, including neonatal abstinence syndrome (NAS). NAS is a withdrawal syndrome experienced by infants shortly after birth. The syndrome most commonly occurs after antenatal exposure to opioids, although other medications have also been implicated (3). From 2000 to 2009, the incidence rate of NAS in Tennessee increased from 0.7 to 5.1 per 1,000 births, exceeding the national average, which increased from 1.2 to 3.4 per 1,000 births (4). NAS is associated with numerous morbidities for the infant, including low birth weight, poor feeding, and respiratory problems (5). Previous population-based analyses of NAS relied on hospital discharge data, which typically become available for analysis only after substantial delay (4,5). In Tennessee, the rising incidence of NAS and its associated public health burden created an urgent need for timelier incidence figures to drive policy and prevention efforts. Beginning January 1, 2013, the Tennessee Department of Health (TDH) made NAS reporting mandatory. A total of 921 cases were reported in 2013 (among 79,954 births), with the most cases clustered in eastern Tennessee; 63% of cases occurred to mothers who were reported to be using at least one substance prescribed by a health care provider (e.g., opioid pain relievers or maintenance medications for opioid dependency), and 33% of cases occurred among women using illicit or diverted substances (e.g., heroin or medications prescribed for someone else). The first year’s surveillance results highlight the need for primary prevention activities focused on reducing dependence/addiction among women of childbearing age and preventing unintended pregnancy among female opioid users.

Beginning in 2012, TDH staff worked with neonatal, obstetrical, and public health stakeholders throughout the state to define the data elements for NAS reporting, with a goal of gathering sufficient data to inform program and policy efforts while minimizing the additional reporting burden on hospitals. Hospitals were advised that typically the diagnosis of NAS involves clinical signs of withdrawal, a history of exposure (prenatal substance use), and evidence of exposure (positive maternal or neonatal drug tests), although not all these elements are absolutely required for reporting the diagnosis. TDH advised hospitals to report cases in which a diagnosis of NAS was assigned to an infant based on a clinical withdrawal syndrome, with symptoms such as feeding difficulty, sleep disturbance, hyperirritability, or seizures. Hospitals were also asked to report data on history of exposure as well as evidence of exposure to support the diagnosis based on clinical signs. An online reporting system allowed for rapid and secure collection of protected health information. Hospitals were asked to report within 30 days of the infant’s diagnosis using a standard set of data fields (Box). Hospitals were introduced to the reporting requirement in many ways, including notification through health care provider organizations, an introductory webinar, and online availability of a reference guide and an FAQ document. On a weekly basis, TDH staff extracted the surveillance data from SurveyGizmo (Widgix, LLC; Boulder, Colorado). Weekly surveillance reports were published online at http://health.tn.gov/mch/nas/nas_summary_archive.shtml.

In 2013, a total of 1,101 cases of NAS were reported through Tennessee’s surveillance system. TDH epidemiologists reviewed the reports and resolved any suspected duplicates with reporting hospital staff. After excluding 39 duplicates and 141 cases without clinical signs consistent with NAS, a total of 921 cases (among 79,954 births) were reported in calendar year 2013 (Table). A provisional comparison of the total number of cases reported during the first 6 months through this surveillance system (N = 426) with counts from hospital discharge data during the same period (N = 488) showed that the surveillance system captured 88.4% of NAS cases identified through administrative claims. In addition to clinical signs of NAS, 98.3% of the cases also had a positive maternal or neonatal drug screen and/or a history of maternal substance use. The highest incidence rate was noted to be in eastern Tennessee, consistent with previous analyses of hospital discharge data. Rates varied across the state health department regions, ranging from 1.6 to 54.2 per 1,000 live births (Figure). More cases of NAS were reported among males compared with females (58.0% versus 41.9%) (p<0.001). The most commonly reported sources of exposure were supervised replacement therapy (such as methadone or buprenorphine, 46.4% of cases), followed by prescription substance obtained without a prescription (40.2%), and nonprescription substance (27.4%). When cases were analyzed using mutually exclusive categories of exposure source, 41.7% of cases involved maternal use of prescription drugs only; another 21.6% involved exposure to at least one drug prescribed by a health care provider and an illicit/diverted drug, and 33.2% involved exposure to illicit/diverted drugs only. A relatively small proportion of cases were reported in which the source of exposure was marked as “no known exposure” (1.4%) or was left blank by the reporting hospital (2.1%) (Table).

BOX **Reporting elements included in a statewide surveillance system for neonatal abstinence syndrome (NAS) — Tennessee, 2013**

**Table:** 

Type of hospitalization – Initial (birth) hospitalization – Transfer from birth facility – Readmission – Diagnosed in outpatient setting Name of birth hospital Name of reporting hospital Last four digits of infant’s hospital chart number Infant’s date of birth Sex of infant – Male – Female – Unknown at time of report Mother’s county of residence Confirmatory drug tests ordered for infant – Hair, pending – Hair, completed – Urine, pending – Urine, completed – Meconium, pending – Meconium, completed – Umbilical cord, pending – Umbilical cord, completed – Other (please specify) Clinical signs of NAS in infant – Yes – No Other supportive elements for diagnosis – Maternal history of using substance known to cause NAS – Positive maternal screening test for substances known to cause NAS – Positive neonatal screening test for substances known to cause NAS Source of substance causing NAS, if known – Maternal, supervised replacement therapy (prescription drug obtained with a prescription) – Maternal, supervised pain therapy (prescription drug obtained with a prescription) – Maternal, therapy for psychiatric or neurologic condition (prescription drug obtained with a prescription) – Maternal, prescription substance obtained without a prescription – Maternal, nonprescription substance – No known exposure but clinical signs consistent with NAS – Other (please specify)

Each week surveillance data were compiled, posted online, and distributed via e-mail to various public health stakeholders across the state, including cabinet-level officials, public health staff, health care providers, reporting hospitals, insurance payers, and community nonprofit agencies. Public health partners (both public and private sector) are using these data to inform local prevention activities. For example, a local health department in one region with high NAS incidence is co-locating family planning services in a methadone clinic, and a large third-party payer is piloting the co-location of substance abuse services in a rural primary care clinic. In addition, TDH is developing automated notifications that will be sent electronically from the state’s prescription drug monitoring program to providers whose patients might be at risk of overdose or other adverse outcomes.

## Discussion

The new Tennessee NAS surveillance system identified a high rate of NAS cases throughout the state (11.6 per 1,000 live births), demonstrating a 16-fold increase in the syndrome since the year 2000. Geographic distribution of cases was skewed with a higher case rate in Tennessee’s eastern, more mountainous counties, consistent with prior analyses showing higher opioid use in those areas compared with other regions of the country (7). Further, the high incidence of NAS in eastern Tennessee counties is consistent with other indicators of opioid use/misuse in Tennessee, including opioid prescriptions and overdose deaths (2,8). Similar to previous population-based analyses (4), the findings in this report indicate that male infants were more likely to be diagnosed with NAS than female infants, suggesting a heightened susceptibility to the syndrome.

The findings in this report are subject to at least three limitations. First, a passive surveillance system might not capture all cases of NAS. However, provisional comparison of counts from this surveillance system with hospital discharge data suggests that Tennessee’s surveillance system captured a majority of cases in real-time and with the advantage of greater detail (e.g., maternal exposure source) compared with administrative claims during its first year of operation. Second, because the system does not gather identifying information, there is a potential for duplicates in reporting. To address this, staff routinely monitor key fields (date of birth, county, sex, and hospital name) to identify possible duplicates; suspected duplicates are then reviewed with the reporting hospital for confirmation. In 2013, a total of 39 cases (3.5%) were found to be duplicates. Finally, the count of NAS depends on accurate diagnosis of the clinical syndrome. There is known variability in the approach to diagnosing NAS, creating the possibility of misclassification (9).

What is already known on this topic?

The incidence of neonatal abstinence syndrome (NAS) has increased substantially across the United States over the past decade, concomitant with an increase in maternal opioid use. Infants with antenatal opioid exposure are more likely to suffer other perinatal morbidities. The high rate of NAS also places a burden on public health and social service systems.

What is added by this report?

In 2013, Tennessee became the first state to establish a public health surveillance system for NAS. In its first year of operation, 921 affected infants were identified, of whom nearly two thirds were born to mothers who used at least one opioid medication prescribed to them by a health care provider during pregnancy. Cases were more common among the Appalachian counties of eastern Tennessee.

What are the implications for public health practice?

Availability of near real-time surveillance data for NAS gives Tennessee actionable data on which to allocate programmatic resources and develop sound public health policy aimed at primary prevention. Inclusion of information on maternal county of residence and source of exposure allows for targeted interventions that best address local needs.

In 63% of NAS cases reported in Tennessee in 2013, the infant was born to a mother who had used at least one substance prescribed to her by a health care provider. This finding is particularly important because health care providers have multiple opportunities to intervene with patients at risk for using substances that could cause NAS. Health care providers can, and in some states, such as Tennessee, are required to use state prescription drug monitoring programs to identify patients with use patterns that increase their risk for dependence/addiction and make appropriate referrals to treatment resources (10).

Targeting prevention of opioid dependence/addiction to women of childbearing age is a primary strategy for preventing NAS and should be considered by states with high rates of the syndrome. Prevention of unintended pregnancy in this population is another primary prevention strategy for NAS. The Tennessee experience of public reporting of NAS suggests that real-time, location-specific data can lead to primary prevention strategies aimed at the most affected populations and also provide a potential mechanism for intervention through health care providers responsible for prescribing substances associated with NAS.

## Figures and Tables

**FIGURE:**
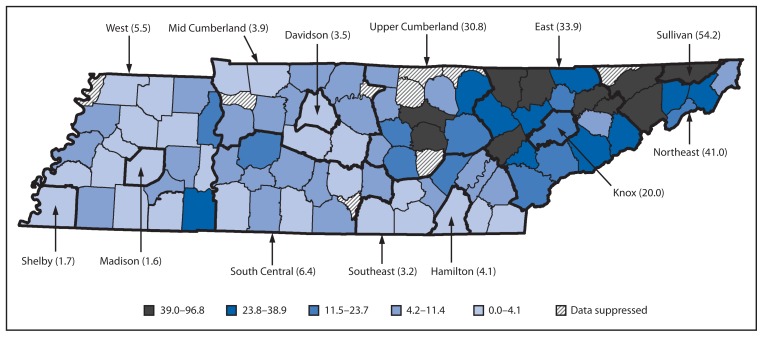
Rate of neonatal abstinence syndrome per 1,000 live births, by mother’s county of residence and state health department region* — Tennessee, 2013 *Thin border lines indicate counties. Thick border lines indicate state health department regions.

**TABLE:** Findings of a statewide surveillance system for neonatal abstinence syndrome (NAS) — Tennessee, 2013

**No. of NAS cases reported**	**921**
**Type of hospital reporting (%)**	
Birth	84.2
Transfer	15.4
Outpatient	0.2
Readmission	0.2
**Sex of infant (%)**	
Male	58.0
Female	41.9
**Source of substance causing NAS (%)**	
Supervised replacement therapy	46.4
Supervised pain therapy	19.0
Therapy for psychiatric or neurologic condition	7.4
Prescription substance obtained without a prescription	40.2
Nonprescription substance	27.4
No known exposure but clinical signs consistent with NAS	1.4
No response (left blank)	2.1
**Elements of case reporting (%)**	
One: clinical signs of NAS only	1.7
Two: clinical signs plus history of exposure only	27.8
Two: clinical signs plus evidence of exposure only	7.7
Three: clinical signs plus history of exposure and evidence of exposure	62.8
